# Holocarboxylase synthetase knockout is embryonic lethal in mice

**DOI:** 10.1371/journal.pone.0265539

**Published:** 2022-04-06

**Authors:** Mahrou Sadri, Haichuan Wang, Toshinobu Kuroishi, Yong Li, Janos Zempleni

**Affiliations:** Department of Nutrition and Health Sciences, University of Nebraska-Lincoln, Lincoln, Nebraska, United States of America; University of Massachusetts Amherst, UNITED STATES

## Abstract

Holocarboxylase synthetase (HLCS) catalyzes the biotinylation of five distinct biotin-dependent carboxylases and perhaps chromatin proteins. HLCS deficiency causes multiple carboxylase deficiency which results in fatal consequences unless patients are diagnosed early and treated with pharmacological doses of biotin. The objective of this study was to develop an HLCS conditional knockout (KO) mouse and assess effects of HLCS knockout on embryo survival. In the mouse, exon 8 is flanked by LoxP sites, thereby removing a catalytically important region upon recombination by Cre. HLCS conditional KO mice were backcrossed for 14 generations with C57BL/6J mice to yield *Hlcs*^*tm1Jze*^. Fertility and weight gain were normal and no frank disease phenotypes and abnormal feeding behavior were observed in the absence of Cre. HLCS knockout was embryonic lethal when dams homozygous for both the floxed *Hlcs* gene and tamoxifen-inducible Cre recombinase (denoted *Hlcs*^*tm1*.*1Jze*^) were injected with tamoxifen on gestational days 2.5 and 10.5. This is the first report of an HLCS conditional KO mouse, which enables studies of the roles of HLCS and biotin in intermediary metabolism.

## 1. Introduction

Holocarboxylase synthetase (HLCS, E.C. 6.3.4.-) is a biotin-protein ligase encoded by a single locus on chromosomes 21 and 16 in humans and mice, respectively [1, 2]. HLCS plays essential roles in intermediary metabolism and in gene repression by epigenetic mechanisms. In intermediary metabolism, HLCS catalyzes the covalent binding of biotin to ε-amino groups in lysine (K) residues in five carboxylases, cytoplasmic acetyl-CoA carboxylase 1, 3-methylcrotonyl-CoA carboxylase, propionyl-CoA carboxylase, pyruvate carboxylase and mitochondrial acetyl-CoA carboxylase 2 [3, 4]. Holo-carboxylases catalyze essential reactions in gluconeogenesis and the metabolism of leucine and fatty acids [5]. Polymorphisms and mutations in the human *HLCS* gene cause a loss in HLCS catalytic activity [6, 7]. Depending on the genetic variation, activity may be rescued by supplementation with pharmacological doses of biotin [7, 8]. If untreated, multiple carboxylase deficiency causes symptoms such as lethargy, hypotonia and metabolic ketolactic acidosis and death in early infancy [9, 10]. No living HLCS null patient has ever been identified, suggesting that loss of HLCS is embryonic lethal [7].

We were first to show that HLCS also catalyzes the covalent binding of biotin to ε-amino groups in K residues in histones H3 and H4 although the abundance of histone biotinylation marks is small and the biological importance of histone biotinylation is controversial [11–16]. Histone modifications play a crucial role in gene regulations via modifying the tightness of chromatin packing, thereby regulating the access to DNA for transcription [17, 18]. As an alternative to direct effects of histone biotinylation on gene expression, it was proposed that HLCS orchestrates the assembly of a multiprotein gene repression complex, composed of EHMT1, DNMT1, MeCP2 and N-CoR [19–21]. Phenotypes of HLCS deficiency included a decrease in heat tolerance, fertility, and overall shortened life span in *D*. *melanogaster* but those reports did not assess whether the phenotypes were due to epigenetic mechanisms or a decrease of carboxylase biotinylation [22, 23].

Progress in the field of HLCS research has been hampered by the absence of a knockout (KO) mouse model. To the best of our knowledge, the only HLCS KO mouse available to date is a non-conditional KO, C57BL/6NJ-C57BL/6NJ-*Hlcs*^*em1(IMPC)J*^/Mmjax, also known as *Hlcs*^*em1(IMPC)J*^ (Mutant Mouse Resource & Research Centers, stock no 42127-JAX). The mouse requires a heterozygote *x* heterozygote mating system and does not allow to exercise spatial and temporal control over HLCS KO. Here we engineered an HLCS conditional KO (CKO) mouse, in which exon 8 (E8) in the *Hlcs* gene is flanked by LoxP sites. E8 is catalytically important based on previous reports that mutations in E8 cause a loss of HLCS activity, therefore deletion of E8 is an appropriate strategy for knocking out HLCS [7]. Exon 8 is present in all three HLCS isoforms reported to date [6]. We donated the HLCS CKO mouse to The Jackson Laboratory and this paper provides important information about the development of the mouse for researchers.

## 2. Materials and methods

### 2.1. Design and assess the efficiency of HLCS KO vector

**Fig 1:**
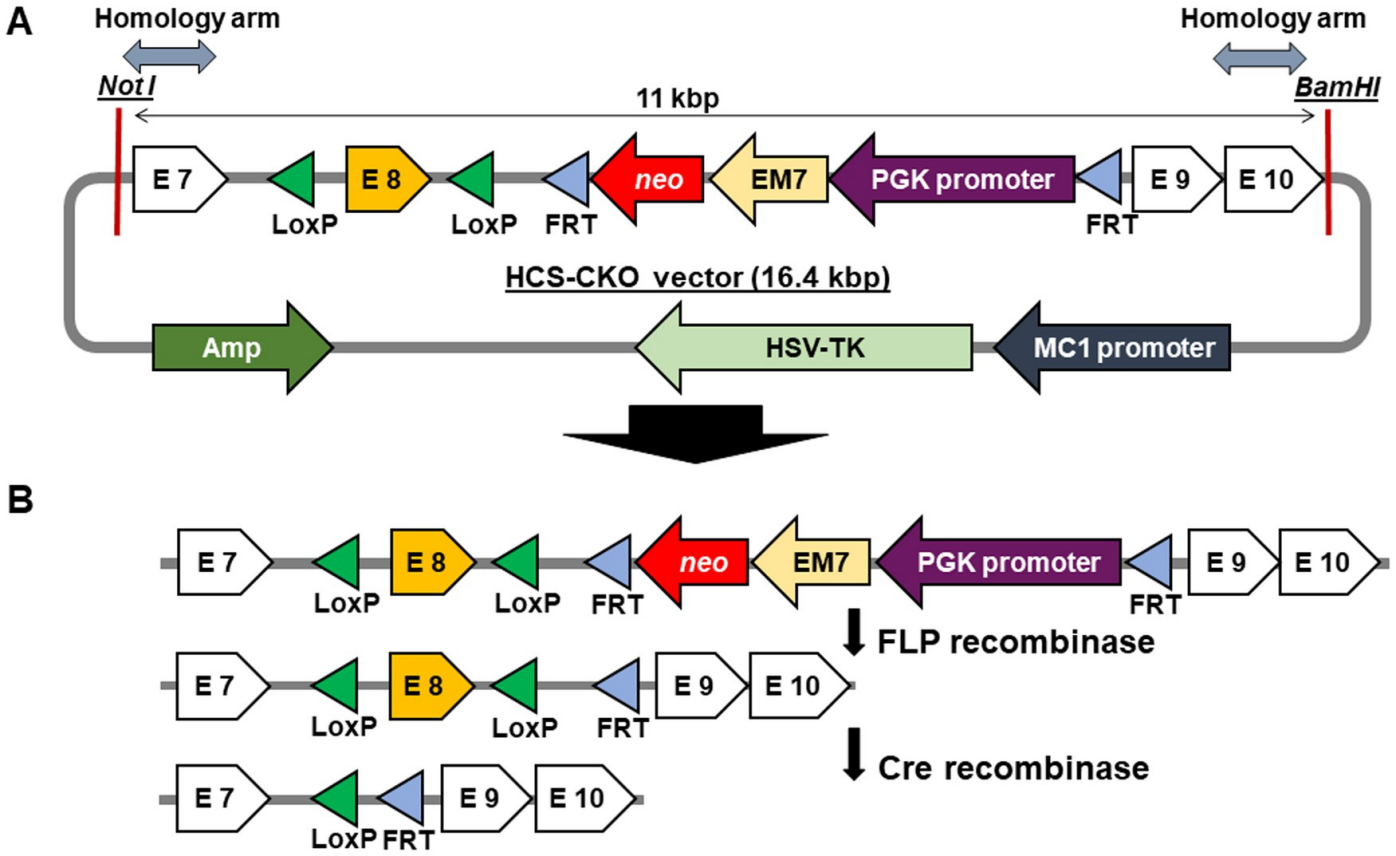
Schematic of the HLCS CKO vector. Amp, ampicillin resistance; E, exon; EM7, prokaryotic promoter; FRT, Flp recognition target; HSV-TK, Herpes simplex virus-1 thymidylate kinase; LoxP, locus of crossover of P1; MC1, target of c-Myc (eukaryotic) promoter; *neo*, neomycin resistance; PGK, phosphoglycerate kinase (eukaryotic) promoter.

The HLCS CKO vector was constructed by using a clone from a BAC library that contained exons 6–11 of the murine *HLCS* gene in 129S1/SvlmJ mice (RP22-406F20, Children’s Hospital Oakland Research Institute). The homology arms facilitate the integration of exon 8 (E8) flanked by LoxP sites and *neo* flanked by FRT sites (Fig 1A). The homology arms flanking the left LoxP site (619 base pairs) and the right FRT site (546 base pairs) facilitate chromosomal recombination and substitution of floxed E8 and *neo* for E8 in the endogenous *Hlcs* gene. Geneticin (G418) and ganciclovir were used as positive and negative selection markers, respectively. Efficacy of HLCS KO was confirmed by transforming *E*. *coli* strain SW106, which expresses arabinose inducible Cre recombinase. Floxed E8 was excised by induction of Cre with 200 μL of 10% arabinose and the HLCS KO vector was purified. The HLCS KO vector was linearized using *ClaI* and used to transfect primary mouse NIH3T3 embryonic fibroblasts by electroporation. Cells were selected in media containing 500 μg/mL G418 (positive selection) and 10 μM ganciclovir (negative selection) for 2 weeks. Surviving clones were isolated by clonal dilution in 96-well plates (theoretically 0.5 cells/well) and subsequently expanded in culture flasks. Transformed clones were screened by PCR and the efficacy of HLCS KO vector was assessed using biotin ligase activity by streptavidin blots [24]. Equal loading was confirmed by using anti-propionyl-CoA carboxylase and anti- pyruvate carboxylase [25].

### 2.2. Engineering a conditional HLCS KO mouse (Hlcs^tm1Jze^)

All animal research was approved by Institutional Animal Care Program at the University of Nebraska-Lincoln and approved the program’s Institutional Animal Care and Use Committee (protocol 1713). In terminal studies mice were anesthetized by using isoflurane and euthanized by a combination of carbon dioxide and cervical dislocation. Mice exhibiting the following signs would have been euthanized prematurely but none of these signs were encountered in our study: weight loss greater than 20%, inability to eat or drink and macroscopically visible tumors. Mice were not restrained beyond normal housing and husbandry procedures.

The HLCS CKO vector was delivered to mouse embryonic stem cells (ES; strain 129ola/E14) by electroporation and 240 clones were screened for genomic integration of FRT-*neo* by using Southern blot analysis. Genomic DNA was digested using *SpeI* and separated on a 0.8% agarose gel and probed with a ^32^P-labeled oligonucleotide, Southern blot probe-F (Table 1) [26]. HLCS-positive ES clones were selected using G418 and injected into blastocysts from hyper ovulating C57BL/6J mice (Jackson stock no. 000664) for subsequent transfer into pseudo pregnant C57BL/6J dams and generation of 129ola/E14—B57BL/6 chimeras [27]. Chimeras were backcrossed with C57BL/6J mice for 10 generations. Germline transmission in chimeras was confirmed by using primers LoxP-F and FRT-R (Table 1) and the following PCR program: denaturation at 94°C for 4 min, 34 amplification cycles (94°C for 30 sec, 57°C for 30 sec, 72°C for 1 min) and a final extension step at 72°C for 10 min. Mice were genotyped by using genomic DNA from toe or tail clips (Azura Mouse Genotyping Kt, Azura Genomics. Inc., cat. no. AZ-1855), PCR primers Floxed-E8-F and Floxed-E8-R (Table 1) and the following PCR protocol: denaturation at 95°C for 30 sec, 34 amplification cycles (95°C for 30 sec, 55°C for 40 sec, 72°C for 1 min) and a final extension step at 72°C for 5 min. Siblings were mated and pups homozygous for floxed E8 were identified in F12 and used to remove the *neo* selection marker and one of the FRT sites (Fig 1B). Briefly, the *neo* marker was removed by mating mice homozygous for floxed E8 with B6.Cg-Tg (Pgk1-flpo)10Sykr/J mice (Jackson stock no. 011065). Removal of the *neo* marker was confirmed by PCR using primers Neo-F and Neo-R (Table 1) and the following PCR protocol: denaturation at 95°C for 30 sec, 34 amplification cycles (95°C for 30 sec, 56°C for 40 sec, 72°C for 1 min) and a final extension step at 72°C for 5 min. Deletion of *neo* was assessed by PCR using primers Flp transgene-F and Flp transgene-R and the following protocol: denaturation at 95°C for 30 sec, 34 amplification cycles (95°C for 30 sec, 56°C for 40 sec, 72°C for 1 min) and a final extension step at 72°C for 5 min. Pups homozygous for floxed E8 in the C57BL/6J background were identified in F14 and denoted *Hlcs*^*tm1Jze*^.

**Table 1:** PCR primers and southern blot probe.

Name	Direction (5´ → 3´)
Floxed-E8-F^1^	ATCCAGCACCAAACAGTGCGAAAC
Floxed-E8-R	GCTCCCAGTGAACAATACCGAAGG
LoxP-F	GACCTGCTTGTCTCCATCTCCAC
FRT-R	TAGTTGCCAGCCATCTGTTGTTT
Flp transgene-F	ATAGCAGCTTTGCTCCTTCG
Flp transgene-R	TGGCTCATCACCTTCCTCTT
Neo-F	GCTGCCATACTTGATCCATG
Neo-R	CTTGGGTGGAGAGGCTATTC
Cre-MUT-F	CTGGCTTCTGAGGACCG
Cre-MUT-R	CCGAAAATCTGTGGGAAGTC
Cre-WT-F	CGTGATCTGCAACTCCAGTC
Cre-WT-R	AGGCAAATTTTGGTGTACGG
E8-F	CATTTGATGTCCTTGGCTGTG
E8-R	AATTAACCAGAACCCCACCG
GAPDH-F	CATCACTGCCACCCAGAAGACTG
GAPDH-R	ATGCCAGTGAGCTTCCCGTTCAG
Southern blot probe-F	CTGGCGGCCTCCAGTTGTCTCGCACAG

^1^F, Forward primer; MUT, mutant; R, reverse primer; WT, wild-type.

### 2.3. Assessment of embryonic lethality of HLCS KO in tamoxifen-inducible HLCS CKO mice (Hlcs^tm1.1Jze^)

*Hlcs*^*tm1Jze*^ mice were bred with tamoxifen-inducible Cre mice (B6;129-Gt(ROSA)26Sor^tm1(cre/ERT)Nat^, Jackson Laboratory stock no 004847). Siblings were mated and pups homozygous for both floxed E8 and Cre were identified by using PCR as described above for E8, and by using primers Cre-MUT-F, Cre-MUT-R, Cre-WT-F, and Cre-WT-R (Table 1) and the following PCR protocol for Cre: denaturation at 95°C for 30 sec, 34 amplification cycles (95°C for 30 sec, 55°C for 40 sec, 72°C for 1 min) and a final extension step at 72°C for 5 min. The mice were denoted *Hlcs*^*tm1*.*1Jze*^ and are in a mixed genetic background (C57BL/6J and 129X1/SvJ). Experienced *Hlcs*^*tm1*.*1Jze*^ breeders were bred overnight and mating was confirmed by the presence of a vaginal plug; wild-type (WT) C57BL/6J breeders were used as controls. On embryonic days 2.5 and 10.5, dams were treated with 200 mg/kg tamoxifen in corn oil (20 mg/mL stock solution) by intraperitoneal injection for three consecutive days [28]. Forty-eight hours after the final injection, dams were euthanized. No embryos were macroscopically visible in *Hlcs*^*tm1*.*1Jze*^ dams injected on embryonic day 2.5 whereas embryos in *Hlcs*^*tm1*.*1Jze*^ dams injected on embryonic day 10.5 had died (see Results). The embryos were used to confirm HLCS KO. Briefly, embryos were flash frozen in liquid nitrogen and kept at -80°C for analysis of HLCS mRNA expression and biotinylation of carboxylases. For analysis of mRNA expression, total RNA was isolated from embryos using the miRNeasy Mini Kit (QIAGEN, Inc., cat. no. 217004) and reverse transcribed by using the QuantiTect Reverse Transcription Kit following the manufacturer’s recommendations (QIAGEN, Inc., cat. no. 205311). Quantitative PCR (qPCR) was performed using primers E8-F and E8-R (Table 1). Data analysis was carried out by the 2^-ΔΔCt^ method using GAPDH as housekeeping gene [29].

For analysis of carboxylase and Hlcs protein, total proteins were extracted from embryos by using RIPA buffer supplemented with proteinase inhibitor cocktail (100-fold final dilution, Thermo Fisher Scientific, Inc., Cat. No. 89900). Protein was quantified by using the Pierce^™^ BCA Protein Assay Kit (Thermo Fisher Scientific, Inc). For analysis of carboxylases equal amounts of protein were separated on 3–8% Tris-acetate gels (40 μg protein/lane) and transblots were probed with anti-pyruvate carboxylase (Proteintech Group Inc, cat. no. 16588-1-AP), anti-propionyl-CoA carboxylase (Proteintech Group Inc, cat. no. 21988-1-AP), anti-acetyl-CoA carboxylase α (Cell Signaling Technology, cat. no. 4190S) and IRDye^®^ 800CW Streptavidin (LI-COR, cat. no 925-32f230). For analysis of Hlcs, equal amounts of protein were separated on 4–12% Bis-Tris gels (40 μg protein/lane) and transblots were probed with anti-HLCS (LSBio, cat. no. LS-C137344). Equal loading was confirmed by probing a housekeeping gene, glyceraldehyde-3-phosphate dehydrogenase (GAPDH). The person analyzing carboxylase expression was blinded regarding genotype. Image Studio^™^ Lite (LI-COR) was used for densitometry analysis.

### 2.4. Statistical analysis

The significance of differences in mRNA expression was assessed by using unpaired t-test. Prism 6 (GraphPad) was used for calculations. The densitometry analysis of immunoblot was conducted using an unpaired t-test (Prism, version 9). Data variances were homogeneous, as confirmed by Bartlett’s test. Data are mean ± standard error of the mean. No data points were removed during analysis. *P* < 0.05 was considered statistically significant.

## 3. Results

### 3.1. Efficacy of HLCS KO vector

**Fig 2:**
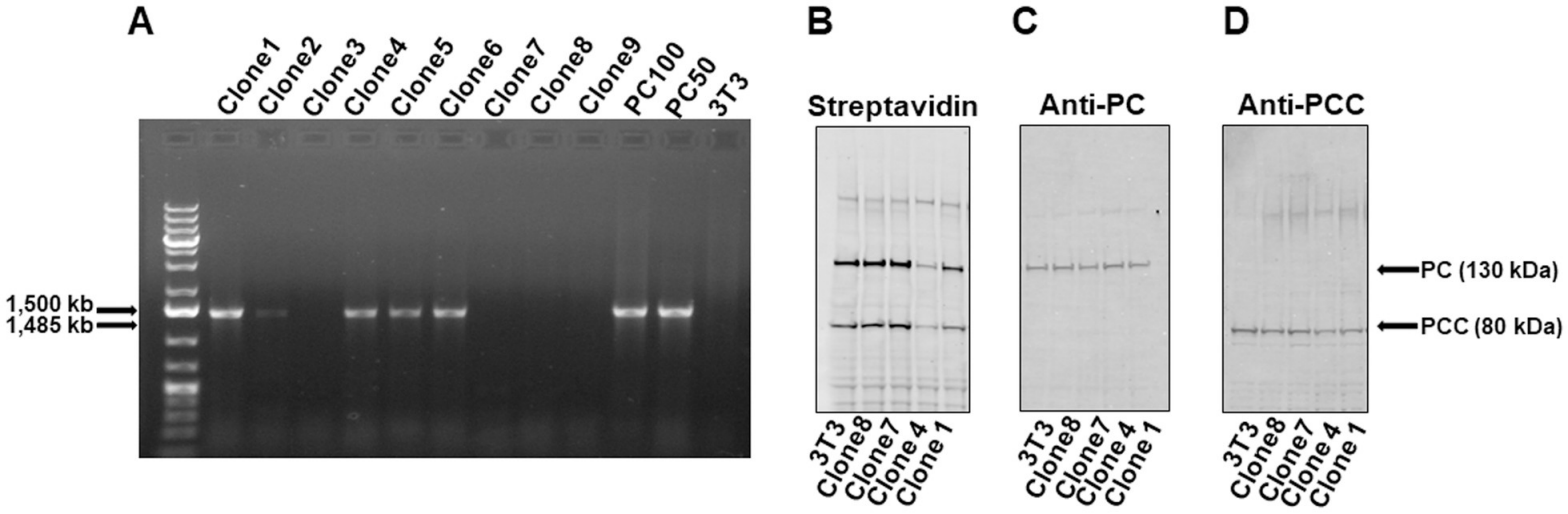
Functionality of HLCS KO in NIH 3T3 mouse fibroblasts. (A) Transformation of cells. Clones were identified by PCR amplification of FloxP-FRT. 3T3 cells transfected with 100 or 50 μg/250 μL of HLCS KO vector before clonal selection; were used as positive controls; wild-type mouse 3T3 embryonic fibroblasts were used as negative control. (B) Carboxylase biotinylation in transformed cells. Biotinylated carboxylases were probed by using streptavidin. (C) Pyruvate carboxylase (PC) in was probed with anti-pyruvate carboxylase. (D) Propionyl-CoA carboxylase (PCC) in was probed with anti- propionyl-CoA carboxylase.

The vector effectively knocked out *Hlcs*. Clones of successfully transformed NIH3T3 fibroblasts were identified by PCR, *e*.*g*., clones 1, 4, 5 and 6 (Fig 2A). As expected, some fibroblasts were not transformed, *e*.*g*., clones 3, 7, 8, and 9. PCR products from using diluted plasmid and untreated fibroblasts were used as template in positive and negative controls, respectively. Successful transformation elicited a loss of biotinylated carboxylases (compare clones 1 and 4 to clones 7 and 8 and untreated fibroblast in Fig 2B). The loss was not due to decreased expression of carboxylases, as evidenced by the expression of apo-pyruvate carboxylase and apo-propionyl-CoA carboxylase (α-chain), which was not different among any of the clones and untreated fibroblasts (Fig 2C and 2D; S1 Raw images).

### 3.2. Engineering HLCS conditional KO mice

**Fig 3:**
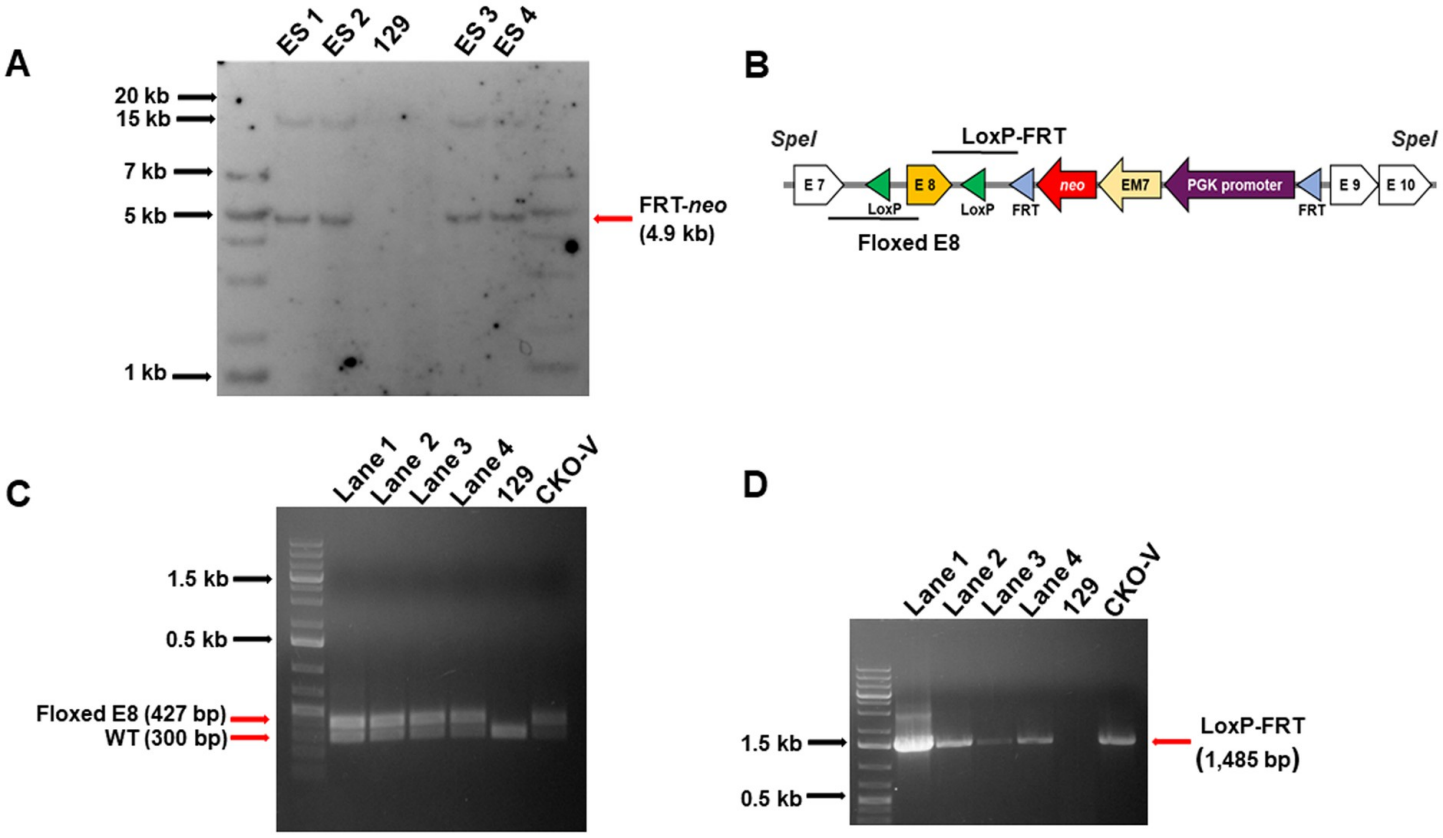
Genomic integration of the HLCS KO vector. (A) Southern blot analysis of ES cells. *SpeI*- and FRT-*neo* digested genomic DNA from transformed ES cells was probed with a ^32^P-labeled oligonucleotide. (B) Schematic of the LoxP-FRT and Floxed E8 amplicons (underlined) in the HLCS KO plasmid used in genotyping. (C) Genotyping floxed E8 in genomic DNA from heterozygous HLCS KO pups (lanes 1–4). “129” denotes genomic DNA from mouse strain 129 (negative control); “CKO-V” denotes a mixture of HLCS KO vectors with and without LoxP sites (positive control). (D) LoxP-FRT sites were probed in genomic DNA. The samples are the same as those used in panel C, including negative and positive controls.

We engineered homozygous HLCS CKO mice by transfection of 129 ES cells and backcrossed mice onto the C57BL/6J genetic background. Transformed ES cells were identified by Southern blot analysis of genomic DNA (Fig 3A). ES2 and ES4 cells were injected into C57BL/6J blastocysts and subsequently implanted in pseudo pregnant dams. Chimeras were backcrossed with C57BL/6J mice for ten generations and floxed E8 and FRT-*neo* fragments were genotyped in each generation by using PCR (Fig 3B). Fig 3C shows representative examples from heterozygous pups in generation F10 and controls. For positive controls we used a mixture of HLCS KO vectors with and without LoxP sites (denoted “CKO-V”). For negative controls, we used genomic DNA from mouse strain 129 (denoted “129”). Germline transmission was also confirmed by presence of LoxP-FRT (Fig 3D).

**Fig 4:**
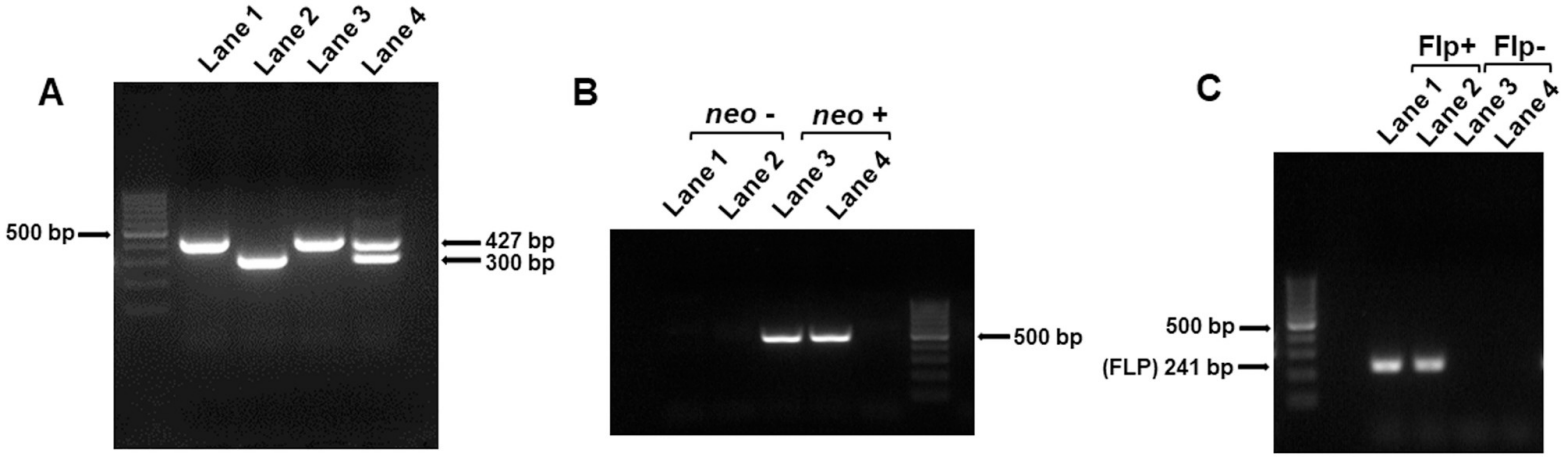
Genomic characterization of *Hlcs*^*tm1Jze*^ mice. (A) Pups homozygous for floxed E8 were identified in generation F12 by using PCR. (B) Identification of pups that tested negative (lanes 1 and 2) and positive (lanes 3 and 4) for *neo*. (C) Identification of pups that tested negative (lanes 3 and 4) and positive (lanes 1 and 2) for Flp. Removal of Flp coincides with loss of *neo* (see panel B). Flp+, presence of Flp recombinase; Flp-, absence of Flp recombinase.

Siblings heterozygous for floxed E8 were mated and homozygous pups were identified by PCR in F12 (Fig 4A, lanes 1 and 3). The *neo* selection marker was removed by mating homozygous mice with Flp mice (Fig 4B, lanes 1 and 2). Deletion of the *neo* marker was also confirmed by detection of Flp in offspring (Fig 4C, lanes 1 and 2). The mice were backcrossed with C57BL/6J mice for a total of 14 generations during this study. The mice were denoted *Hlcs*^*tm1*.*1Jze*^. To date, *Hlcs*^*tm1Jze*^ mice did not show signs of frank disease phenotypes or abnormal behavior. The mice have a normal feeding behavior, weight gain and fecundity. We kept *Hlcs*^*tm1*.*1Jze*^ mice until age four months when they were replaced with younger breeders. Mice were kept in a 12-hour light/dark cycle at 22°C in a climate-controlled environment. Mice were provided with enrichment toys and whenever possible housed in groups of five animals per cage. Mice were monitored daily for macroscopically visible tumors, failure to thrive and signs of aggression; no such signs were observed.

### 3.3. Embryonic lethality of HLCS KO

**Fig 5:**
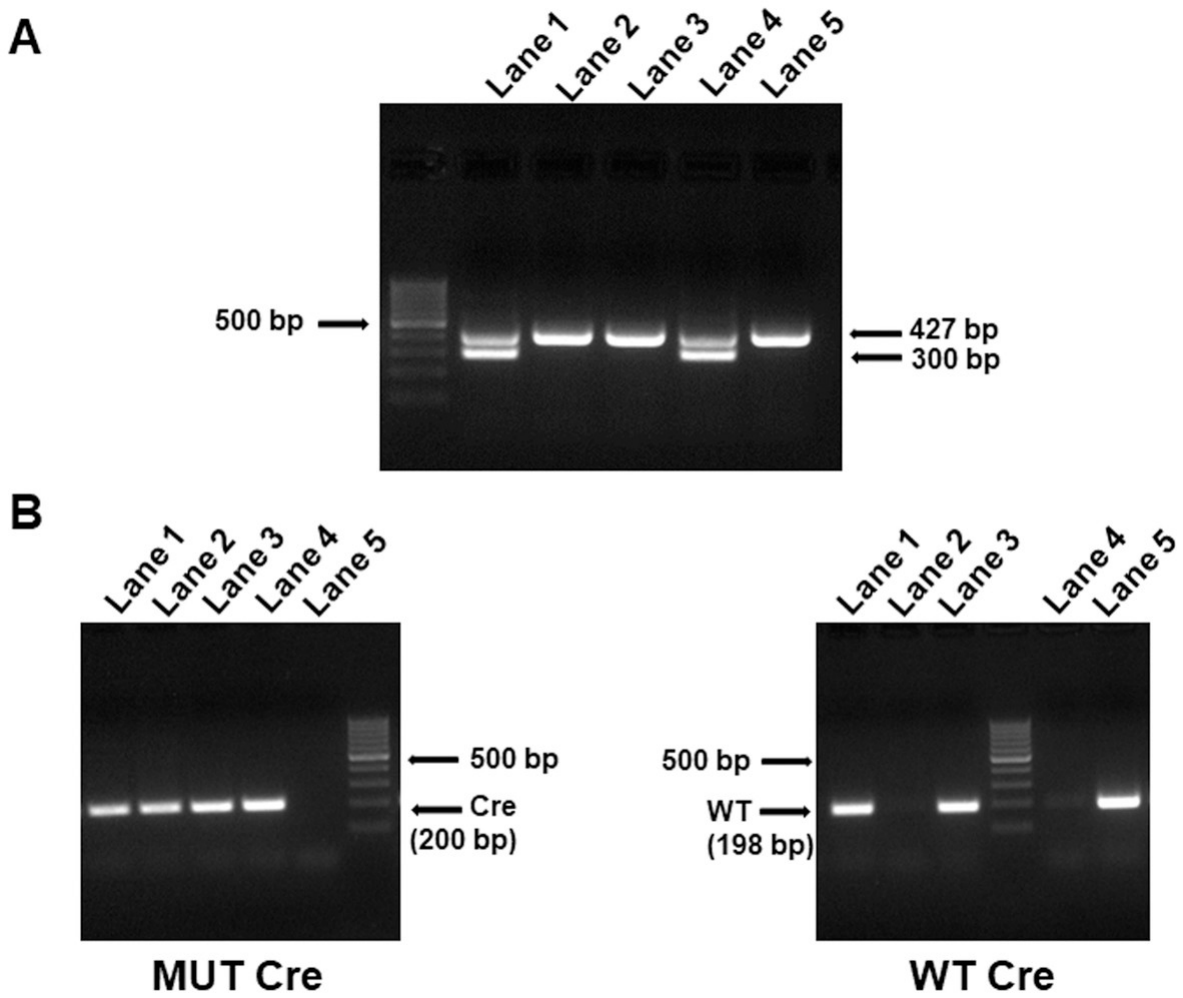
Floxed E8 and tamoxifen inducible Cre recombinase in *Hlcs*^*tm1*.*1Jze*^ mice. (A) Pups homozygous for floxed E8 (lanes 2, 3 and 5) and heterozygous for floxed E8 (lanes 1 and 4) were identified by PCR. (B) Pups homozygous for Cre recombinase (lanes 2 and 4) and heterozygous for Cre recombinase (lanes 1, 3 and 5) were identified by PCR. Lanes with the same labels represent DNA from the same mouse across the gels shown. MUT Cre, mutant Cre recombinase allele; WT Cre, wild-type Cre recombinase allele.

**Fig 6:**
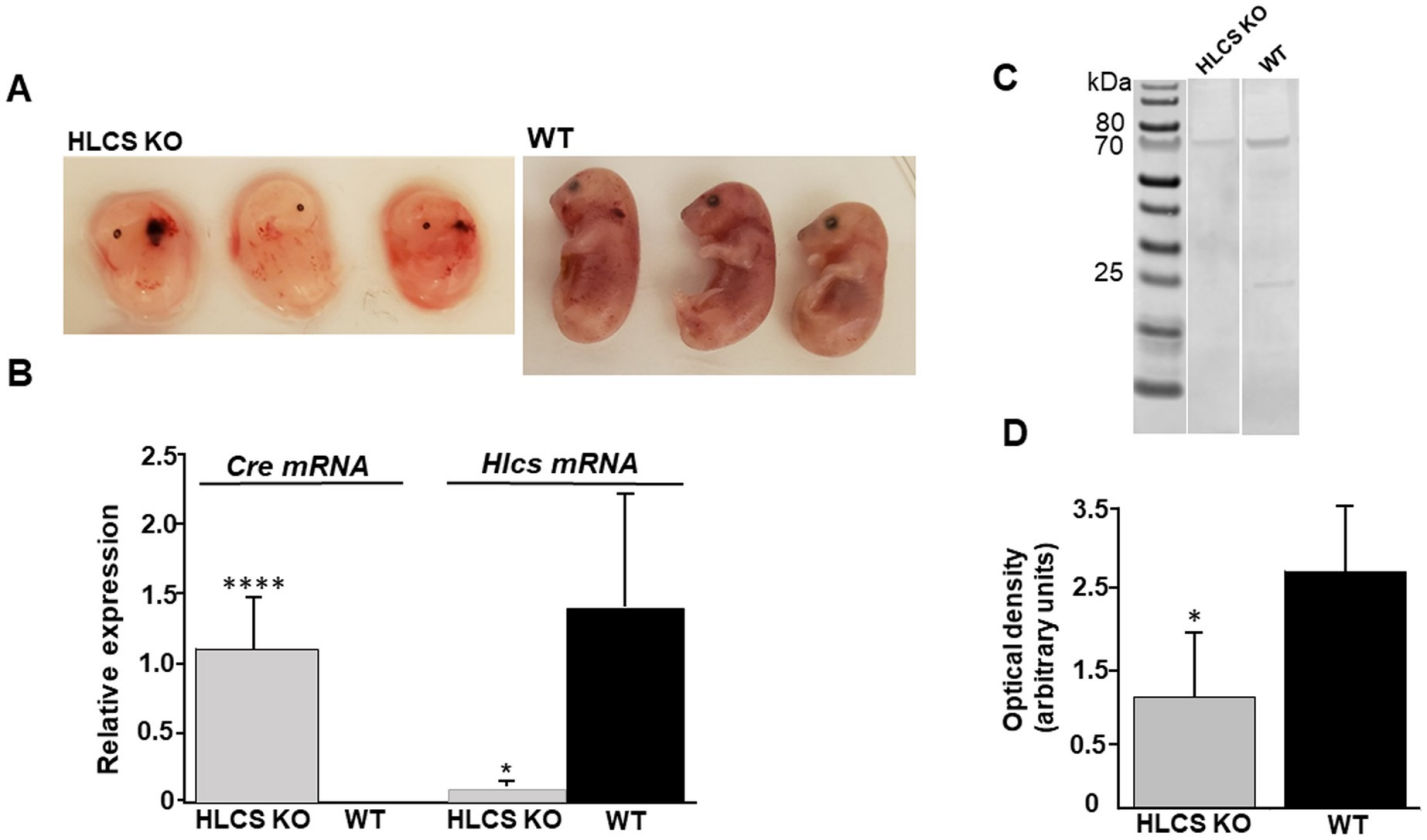
Embryonic development in *Hlcs*^*tm1*.*1Jze*^ mice. (A) Embryos were dissected from *Hlcs*^*tm1*.*1Jze*^ and WT dams 2 days after the final tamoxifen injection on embryonic day 14.5. (B) Expression of *Cre* mRNA and *Hlcs E8* mRNA in *Hlcs*^*tm1*.*1Jze*^ embryos. (C and D) Expression of Hlsc at protein level in Hlcstm1.1Jze embryos. *P < 0.05 *vs*. WT; ****P < 0.0001 *vs*. WT; n = 3. Values are means ± standard error of the mean.

HLCS KO is embryonic lethal. *Hlcs*^*tm1Jze*^ mice were mated to B6;129-Gt(ROSA)26Sor^tm1(cre/ERT)Nat^. Siblings (F1) were mated and pups homozygous for both floxed E8 and Cre recombinase were identified by PCR after two generations of mating (Fig 5, lane 2). Homozygous mice were mated, and tamoxifen was administered for three consecutive days starting on embryonic days 2.5 or 10.5. Dams were euthanized 48 hours after the final injection. No embryos were observed in HLCS KO dams treated with tamoxifen on embryonic days 2.5–4.5 (not shown). In contrast, embryos were observed in HLCS KO dams treated with tamoxifen on embryonic days 10.5–12.5. While the average number of embryos (6, 8 and 9 pups per litter; mean ± SEM = 7.7 ± 0.9 per dam) was the same as in C57BL/6J controls (6, 8 and 8 pups per litter; mean ± SEM = 7.3 ± 0.7 per dam; n = 3), the embryos in HLCS KO dams had ceased to grow (0.19 ± 0.04 g/pup compared to 0.66 ± 0.05 g/pup in controls; n = 3) and died. *Hlcs*^*tm1*.*1Jze*^ embryos displayed hemorrhagic spots and limb shortening, which was not observed in WT embryos (Fig 6A). HLCS KO embryos expressed Cre recombinase mRNA, whereas the expression of Hlcs mRNA was only 4.1 ± 3.5% of that in WT controls (Fig 6B). The substantial decrease in mRNA expression was associated with a decrease in Hlcs protein expression. The level of Hlcs protein decreased by 53% in HLCS KO embryos compared to WT embryos within 48 hours of the last dose of tamoxifen (Fig 6C and 6D).

**Fig 7:**
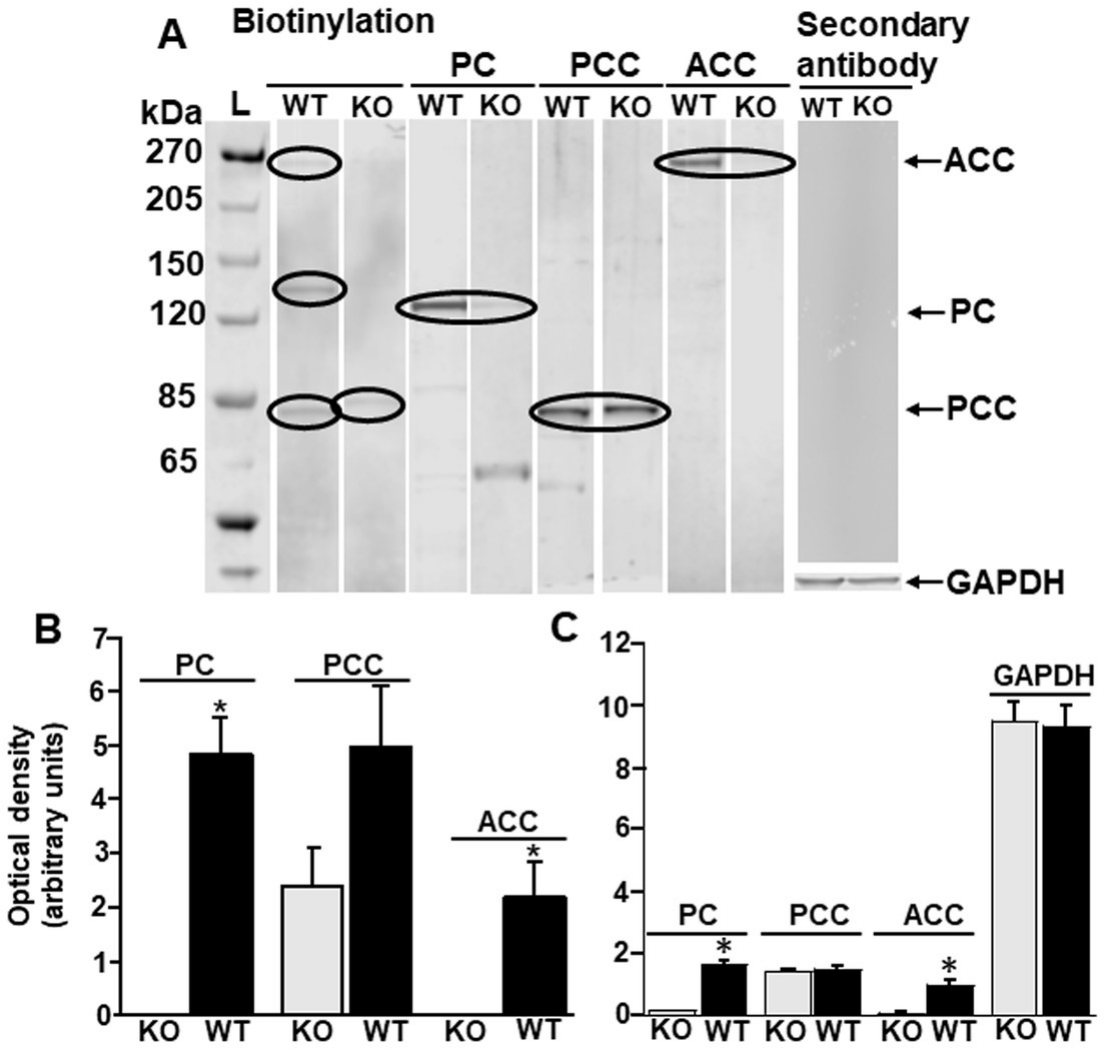
Expression of apo-carboxylases and holo-carboxylases in tissue from HLCS knockout (KO) and wild-type (WT) embryos. (A) Steptavidin blots (“biotinylation”) and immunoblots of carboxylases in HLCS KO and WT embryos. The lanes to the far right depict transblots probed with secondary antibody only (top) and anti-GAPDH (bottom). (B) Densitometry analysis of holocarboxylases probed with streptavidin. (C) Densitometry analysis of carboxylase expression probed with anti-PC, anti-PCC and anti-ACC. Note that the antibodies detect both biotinylated and non-biotinylated carboxylases. Data were presented as mean ± SD (*P < 0.05 vs control, n = 3). KO, HLCS knockout; WT, HLCS wild-type.

HLCS KO caused a decrease in carboxylase biotinylation. Biotinylated PC and ACC were barely detectable in KO embryos whereas the more than 50% decrease in PCC biotinylation was not statistically significant (Fig 7A and 7B). Secondary antibody only produced no signal. The expression of (non-biotinylated) apo-carboxylase protein followed the same pattern (Fig 7C). HLCS KO caused a decrease in the abundance of apo-PC and apo-ACC protein, whereas the expression of apo-PCC was not different in HLCS KO and WT embryos. Equal loading was confirmed by using anti-GAPDH.

## 4. Discussion

To the best of our knowledge this is the first report of an HLCS CKO mouse, *Hlcs*^*tm1Jze*^. Strengths of the *Hlcs*^*tm1Jze*^ mouse include that is in the C57BL/6J background and the absence of disease phenotypes and aberrant behavior, and normal life span and fecundity. While most of these strengths also apply to the new strain, *Hlcs*^*tm1*.*1Jze*^ which expresses tamoxifen-inducible Cre recombinase, strain *Hlcs*^*tm1*.*1Jze*^ is on a mixed genetic background. Unlike the non-conditional HLCS KO strain, *Hlcs*^*em1(IMPC)J*^ in the Mutant Mouse Resource & Research Centers, *Hlcs*^*tm1*.*1Jze*^ allows to exercise spatial and temporal control over HLCS KO, e.g., for studies of HLCS-dependent catalysis in embryonic development [30].

As expected, HLCS KO was embryonic lethal in both early stages and midway through pregnancy. We propose that lethality was due to a depletion of biotinylated carboxylases. Embryonic lethality of HLCS KO is consistent with previous reports suggesting that dietary depletion of the HLCS substrate, biotin is teratogenic in rodents [31, 32]. Future studies should further enhance rigor and reproducibility by mating heterozygous KO mice and assessing the Mendelian genotype distribution.

We conducted an *in silico* analysis of recombination events. Removal of exon 8 causes a frame shift which, theoretically, leads to the expression of an unnatural transcript coding for a protein of 527 amino acids. We do not know if the transcript was spliced and translated into protein.

It is worthwhile discussing two limitations of the HLCS KO mouse developed here. First, the mouse does not permit to selectively knock out HLCS with translational start sites in methionine residues 1, 7 and 58 in exons 6 and 7 [1]. This limitation impedes studies of the variant with a start site in methionine-58 which is enriched in the nuclear compartment [15, 22]. If so desired, further advances in HLCS KO studies in mice will need to mutate ATG start codons in analogy to our previous studies in cell cultures [15] probably by using CRISPR/Cas technology. Second, it is unknown why we detected residual levels of biotinylated carboxylases in NIH 3T3 fibroblast and HLCS KO embryos. We speculate that holo-carboxylases were synthesized prior to knocking out HLCS. For example, the half-lives of rat liver holo-PC and holo-ACC1 have been estimated to be 4.6 days h and 48–59 hours, respectively, and the half-life of holo-PC was 28–35 hours in the mouse preadipocyte cell line, 3T3-L1 [33–35]. There is the possibility that a tamoxifen-inducible (HLCS WT) mouse might have been a better control than a C57BL/6J mouse. The carboxylase biotinylation signal was stronger in NIH3T3 fibroblasts compared to mouse embryos in this study. We speculate that the differences were caused by tissue-specific differences in carboxylase synthesis and turnover. For example, the expression of ACC1 protein is more than four times higher in the human liver compared human soft tissue [36]. Third, homozygous tamoxifen-inducible Cre mice (as opposed to C57BL/6J mice) might be a good, if not better, choice for a control in future studies.

To date, the molecular mechanisms underlying the role of HLCS and biotin depletion in embryonic development are unknown. We conclude that the availability of an HLCS CKO mouse will facilitate such studies, *e*.*g*., by comparing effects of maternal and embryonic loss of HLCS. HLCS KO mice have been donated to the Jackson Laboratories repository for unrestricted access by the biotin research community.

## Supporting information

S1 Raw images Raw images of the original gels used to assemble Figs 2, 6C and 7A. (A) NIH 3T3 mouse fibroblasts probed with streptavidin, anti-pyruvate carboxylase and anti-propionyl-CoA carboxylase. (B) Protein extracts from mouse embryos probed with anti-HLCS, anti-PC, anti-PCC, anti-ACC and streptavidin-conjugated IRDye^®^ 800CW. ACC, acetyl-CoA carboxylase α; HLCS, holocarboxylase synthetase; KO, HLCS knockout; PC, pyruvate carboxylase; PCC, propionyl-CoA carboxylase; WT, HLCS wild-type.
(TIF)

## Abbreviations

ACC: acetyl-CoA carboxylase α
CKO: conditional knockout
HLCS: holocarboxylase synthetase
KO: knockout
MUT: mutant
PC: pyruvate carboxylase
PCC: propionyl-CoA carboxylase
WT: wild-type
