# Supplementary figures and images for: Holocarboxylase synthetase knockout is embryonic lethal in mice
Source: PLoS One. 2022 Apr 6;17(4):e0265539. doi: 10.1371/journal.pone.0265539 (PMC8985998; doi:10.1371/journal.pone.0265539)

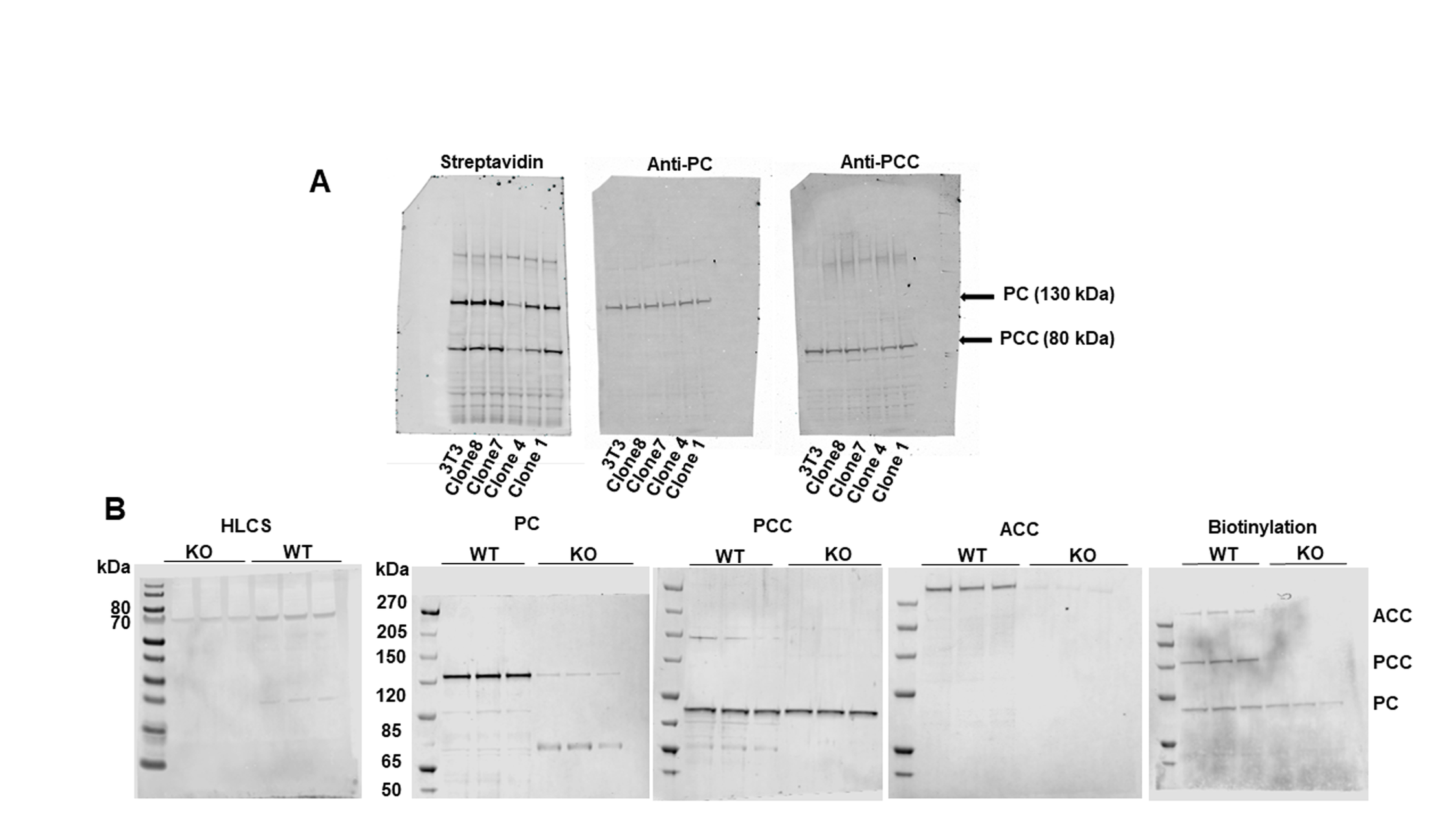

Supplement: S1 Raw images — (A) NIH 3T3 mouse fibroblasts probed with streptavidin, anti-pyruvate carboxylase and anti-propionyl-CoA carboxylase. (B) Protein extracts from mouse embryos probed with anti-HLCS, anti-PC, anti-PCC, anti-ACC and streptavidin-conjugated IRDye® 800CW. ACC, acetyl-CoA carboxylase α; HLCS, holocarboxylase synthetase; KO, HLCS knockout; PC, pyruvate carboxylase; PCC, propionyl-CoA carboxylase; WT, HLCS wild-type. (TIF) [file pone.0265539.s001.tif]
